# From Failure to Positive Result: Turning Aortic Insufficiency in Neocuspidization Procedure Into Excellent Results

**DOI:** 10.1016/j.cjco.2025.02.013

**Published:** 2025-02-20

**Authors:** Marien Lenoir, Loïc Mace, Beatrice Desnous, Grégoire Stolpe, Jean-Marc El Arid

**Affiliations:** a Department of Pediatric Cardiac Surgery, Hôpital Timone Enfant, AP-HM, Aix Marseille University, Marseille, France; b Department of Pediatric Neurology, Hôpital Timone Enfant, AP-HM, Aix Marseille University, Marseille, France; c Department of Adult Cardiology, Hôpital Timone Enfant, AP-HM, Aix Marseille University, Marseille, France; d Department of Pediatric Cardiac Surgery, Hôpital Tours, Tours, France

**The neocuspidization technique is a surgical procedure used for aortic valve replacement. The technique involves reconstructing the aortic valve using autologous pericardium treated with glutaraldehyde. The results are excellent, with no need for long-term anticoagulation, a maximal effective orifice area, excellent survival, and freedom from reoperation. However, the technique requires expertise, particularly in calibrating the size of the cusps; a meta-analysis reports immediate complications requiring aortic valve replacement in proportions ranging from 0.7% to 2.2%.** **^1^** **We describe a case of failure of the neocuspidization procedure that required a second aortic clamping to add an annuloplasty that resulted in excellent outcomes.**

## Case

**Figure 1:**
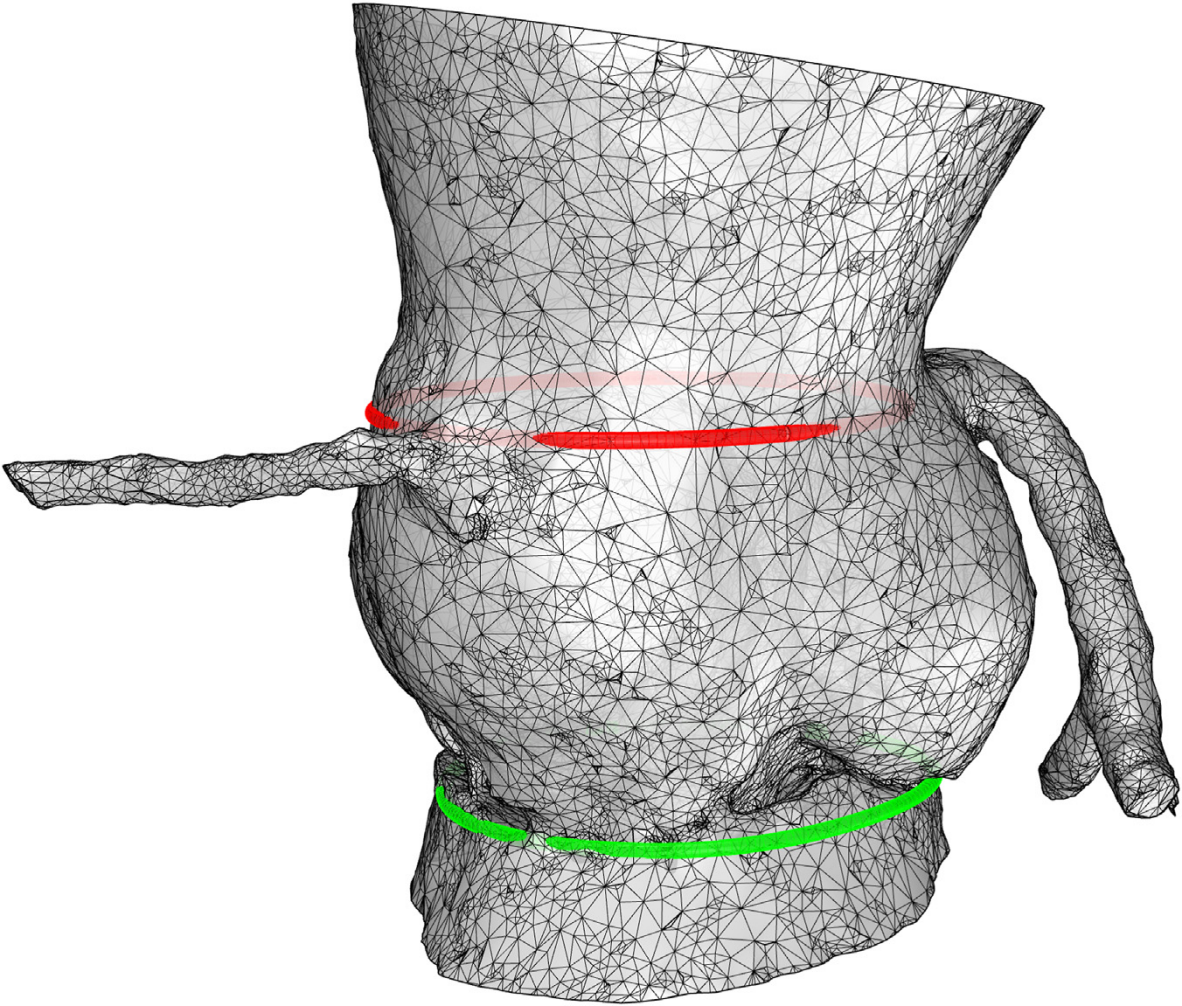
A coronary computed tomography scan showed an aortic basal ring (**green**, 32.4 mm) and the sinotubular junction (**red,** 35.6 mm).

A 23-year-old man, followed since birth for bicuspid aortic valve, presented with complications including severe aortic insufficiency and dilation of the ascending aorta. The patient remained asymptomatic. Echocardiography revealed a unicuspid aortic valve, accompanied by severe insufficiency, with a regurgitant orifice area of 0.3 cm^2^, and a regurgitant volume of 80 mL. Additionally, aortic stenosis was present, with maximal and mean gradients of 30 mm Hg and 19 mm Hg, respectively. The left ventricle showed signs of dilation (left ventricular end-systolic diameter = 54 mm), yet the ejection fraction remained at 68%, and no shunt was detected. Preoperatively, a coronary computed tomography scan showed left ventricular hypertrophy with an aneurysm of the ascending aorta (48 mm), sino-tubular junction (35.6 mm), and aortic annulus (32 mm; Fig. 1).

**Figure 2:**
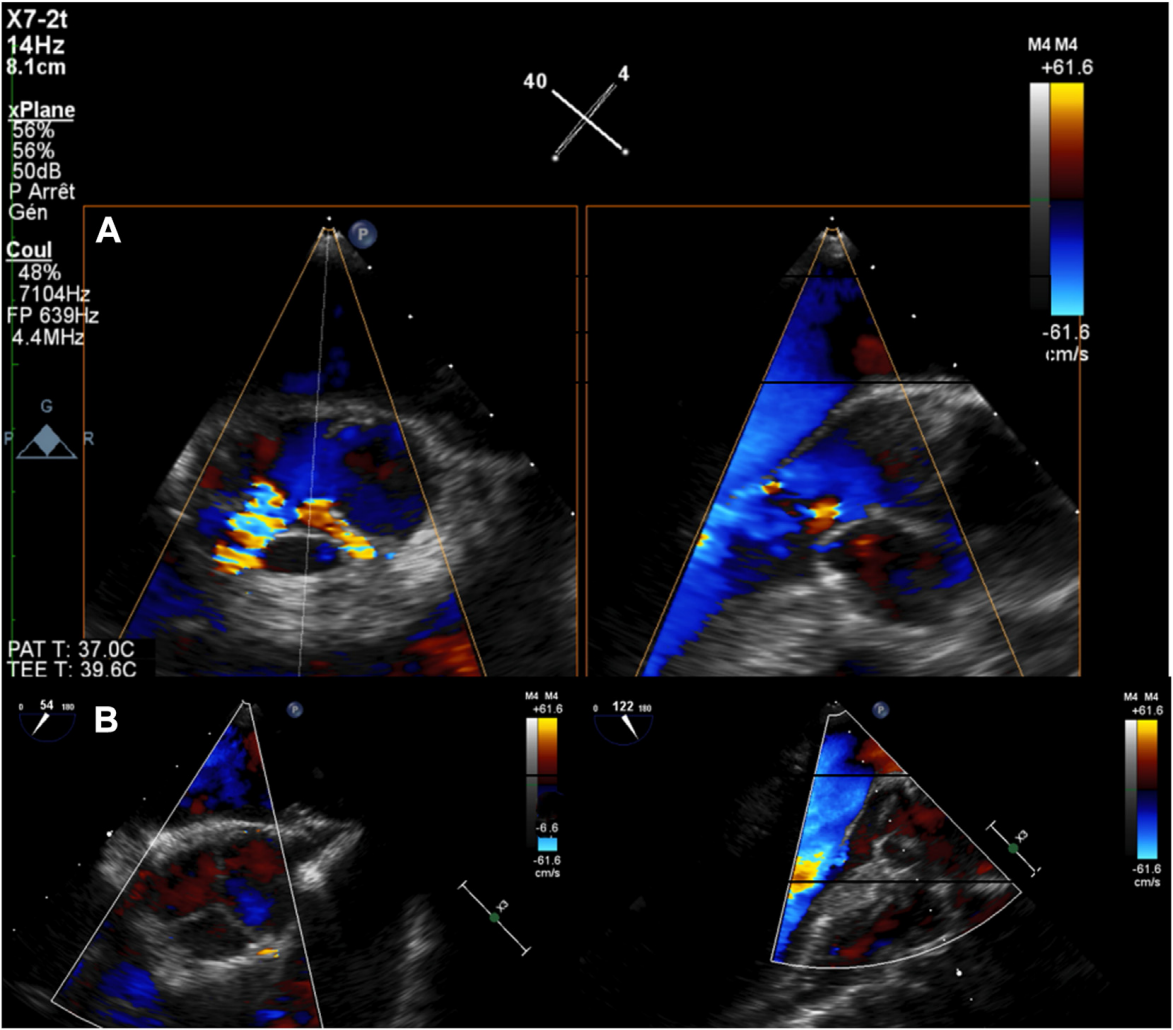
(**A**) Postoperative transesophageal echocardiography revealed moderate aortic insufficiency after the first aortic cross-clamping. (**B**) Shown is trivial aortic regurgitation after the second aortic cross-clamping.

Following a median sternotomy and pericardium harvesting, cardiopulmonary bypass, cross-clamping, and selective cardioplegia were employed. Intraoperatively, a unicuspid aortic valve with a functional commissure between the left coronary and noncoronary cusps, along with 2 raphes, was discovered. The unicuspid valve was excised, and sizers were utilized to determine inter-commissural distances. We found a size of 31 mm for all 3 cusps. We cut the tanned autologous pericardium patch into 3 cusps, each measuring 31 mm, with a perfectly equivalent distribution of the leaflets, with commissural orientation close to 120° (ie, 120° for the noncoronary sinus, 126° for the left coronary sinus, and 120° for the right coronary sinus). Then, the ascending aorta was replaced with a 30-mm Dacron graft, and sinus rhythm resumed once the aortic clamp was removed. Postoperative transesophageal echocardiography revealed no abnormalities, except for moderate aortic insufficiency at the noncoronary/left-coronary neocommissure, without prolapse (Fig. 2A; Video 1 , view video online), and a 31-mm aortic annulus was observed.

A second aortic clamping was performed, accompanied by subcoronary annuloplasty as described by Youssefi et al.,^2^ utilizing a 30-mm Dacron graft. Subsequent echocardiography showed a 25-mm aortic annulus, with trivial aortic regurgitation and a mean aortic gradient of 7 mm Hg (Fig. 2B; Video 2 , view video online).

Operative times for the first and second cross-clamps and cardiopulmonary bypass were 90 and 106 minutes, and 47 and 60 minutes, respectively. The patient experienced an uneventful recovery, spending 3 days in the intensive-care unit, and 8 days total in hospitalization. Rehabilitation progressed smoothly, and the patient was discharged with a prescription for Kardegic 75 mg for 6 months.

The transthoracic echocardiogram performed at 6 months shows a nondilated, non-hypertrophied left ventricle, with preserved systolic function (left ventricular ejection fraction = 66%). The Ozaki aortic valve had a low mean gradient (6 mm Hg), with a coaptation height of 19 mm and a trivial regurgitation.

## Discussion

The surgical intervention performed on our patient involved replacing the aortic valve using the Ozaki technique, followed by subcoronary annuloplasty. Immediate results showed moderate aortic regurgitation, which was reduced to a trivial level with a low mean aortic gradient after the second cross-clamping was performed. Despite the prolonged aortic clamping times (90 minutes during the first clamping and 47 minutes during the second), no complications or cardiac dysfunction occurred.

The Ozaki technique has shown promising results in the literature, with high success rates (no surgery to convert to a prosthetic valve replacement was required in Ozaki et al.^3^) and good midterm durability (the cumulative incidence of reoperation was 4.2% at 118 months).^3^^,^^4^ However, complications, such as the need for a second intervention to reduce aortic regurgitation, commonly are unreported. A meta-analysis reports immediate complications requiring aortic valve replacement in proportions ranging from 0.7% to 2.2%.^1^

The main complication encountered during the intervention was persistent aortic regurgitation after the initial repair. The first reason for this complication was that the pericardial patches were undersized and poorly calibrated, compared to what was needed. This occurrence underscores the importance of using oversized rather than undersized patches, as described in the meta-analysis.^1^ The second reason is that the sinotubular junction measured 30 mm, with an aortic ring of 31 mm, resulting in an aortic annulus and sinotubular junction ratio of 1 after the first clamping. By adding an additional anatomic ring, the ratio became 25/30 or 0.83, which was closer to the physiological ratio.

Our strategy of annuloplasty was adopted because we could not perform a re-neocuspidization, due to the lack of available autologous pericardium. By adding subcoronary annuloplasty, we were able to eliminate the leak without causing a significant increase in the aortic gradient. This proactive management of the complication resulted in a favourable outcome, demonstrating the effectiveness of subcoronary annuloplasty in this context.

We can draw a parallel with the case described in El-Eshmawi et al.,^5^ which highlights the importance of the second cross-clamping to achieve a perfect result after mitral repair. This article also shows that the second cross-clamping does not significantly increase the incidence of morbidity and mortality. Finally, we believe that we could have added annuloplasty during the first clamping to help stabilize the aortic annulus, as we do in the Ross procedure.

### Conclusion

This case underscores the importance of precisely calibrating cusp size during the neocuspidization procedure, as undersized patches can lead to persistent regurgitation postoperatively. In such cases, subcoronary annuloplasty is a valuable adjunctive strategy, especially in patients with a dilated aortic ring, offering the potential to mitigate residual regurgitation while avoiding valve replacement.

**Novel Teaching Points**

• Precise cusp-size calibration during the neocuspidization procedure is of utmost importance.
• Subcoronary annuloplasty has proven to be a valuable adjunctive strategy in addressing immediate aortic regurgitation following the Ozaki procedure.

## Supplementary Material

Video 1 Postoperative transesophageal echocardiography (TEE) revealed moderate aortic insufficiency after first aortic cross clamp.

Video 2 TEE showed trivial aortic regurgitation after second aortic cross clamp.
